# Effects of fire dragon cupping therapy for patients with post-stroke shoulder-hand syndrome: a systematic review and meta-analysis

**DOI:** 10.3389/fneur.2026.1857245

**Published:** 2026-07-30

**Authors:** Yiqiang Xu, Hongman Shen, Yue Yang

**Affiliations:** 1 Heilongjiang University of Chinese Medicine, Harbin, Heilongjiang, China; 2 First Affiliated Hospital, Heilongjiang University of Chinese Medicine, Harbin, Heilongjiang, China

**Keywords:** fire dragon cupping, reflex sympathetic dystrophy, shoulder-hand syndrome, stroke, traditional Chinese medicine

## Abstract

**Background:**

Shoulder-hand syndrome (SHS), as a significant complication of stroke, has seen a continuous increase in incidence. The Fire Dragon Cupping (FDC) therapy is an emerging cupping therapy (CT), and this study aims to summarize the clinical evidence of FDC therapy intervention for SHS, providing guidance for its application and development.

**Methods:**

We searched 11 databases from inception to December 12, 2025. Data were pooled using standardized mean differences (SMD) and risk ratios (RR) with 95% confidence intervals (CIs) and 95% prediction intervals (PIs). To explore high heterogeneity, we prespecified subgroup analyses, univariate meta-regression, and Trial Sequential Analysis (TSA). Risk of bias and certainty of evidence were assessed using ROB-2 and the GRADE framework, respectively.

**Results:**

A total of 11 Randomized Controlled Trials (RCTs) were included in this study, involving 1,087 patients. Notably, all these studies were from China. Pooled estimates indicated FDC might improve clinical efficacy (RR = 1.20, 95% CI:[1.12 to 1.28], 95% PI: [1.10–1.31], *p* < 0.0001, TSA confirming sufficient cumulative sample size, required information size (RIS) = 267) and upper motor function (SMD = 1.01, 95% CI:[0.76 to 1.26], 95% PI: [0.21 to 1.80], *p* < 0.0001). For hand swelling (SMD = −1.34, 95% CI:[−1.98 to −0.70], 95% PI: [−3.59 to 0.91], *p* < 0.0001, I^2^ = 91.9%), and pain intensity (SMD = −1.57, 95% CI:[−2.15 to −0.98], 95% PI: [−3.74 to 0.60], *p* < 0.0001, I^2^ = 92.3%), heterogeneity was high and its sources were not identified. Given the prediction intervals that cross the null, the pooled estimates for hand swelling and pain intensity are reported for transparency only and should not be interpreted as summary effects, in line with the cautious reading in the body.

**Conclusion:**

The apparent therapeutic benefits must be interpreted with extreme caution due to unresolvable severe heterogeneity, inherent lack of blinding, disparate control interventions, and limited generalizability due to an exclusive reliance on Chinese-language literature. Current evidence is insufficient to formulate strong clinical recommendations, future multi-center, adequately blinded RCTs are mandated.

## 1 Introduction

Shoulder-hand syndrome (SHS) is also known as reflex sympathetic dystrophy (RSD) (1). It is a highly prevalent and disabling complication following a stroke (2). The International Association for the Study of Pain (IASP) currently categorizes this condition as type I complex regional pain syndrome (CRPS-I) (3). It typically develops 1–3 months post-stroke. This timeframe is a critical phase for patient rehabilitation. The incidence rate among stroke survivors is reported to be 49% (2).

A recent retrospective review identifies the primary symptoms of SHS. These include pain, swelling, and motor dysfunction in the affected shoulder and hand (4). Without prompt intervention, patients in advanced stages experience muscle and joint atrophy alongside irreversible disability (5). Furthermore, SHS is a chronic and persistent condition. It imposes long-term financial and psychological burdens on patients. Research indicates that 31% of individuals are unable to return to work within 2 years of disease onset (6). This leads to a substantial increase in medical expenses. Additionally, the prevalence of anxiety and depressive symptoms among these patients rises significantly (6).

Despite extensive research, the exact pathological mechanism of SHS remains unclear (1). Most studies closely link its onset to post-traumatic inflammation. Elevated levels of TNF-α, IL-6, IL-8, and substance P are considered the primary drivers (5). Additionally, central sensitization plays a significant role in the development of this condition.

Currently, there is no definitively effective management protocol for SHS patients. Guidelines recommend a multidisciplinary approach combining pharmacological and non-pharmacological treatments (6). Physical rehabilitation is the preferred conservative therapy. However, some studies indicate its clinical benefits are limited. Pharmacological treatments often involve nonsteroidal anti-inflammatory drugs and opioids (2). These medications carry significant side effects and are unsuitable for long-term use. Moreover, evidence supporting their overall efficacy remains controversial (4). Therefore, there is an urgent need to identify novel interventions with long-lasting therapeutic effects.

The application of Traditional Chinese Medicine (TCM) for SHS patients has increased in recent years (6). Common therapies include cupping therapy (CT), moxibustion, and tuina. These interventions demonstrate favorable clinical efficacy and low medical costs (7, 8). As a result, they provide new complementary and alternative treatment options for SHS patients.

CT has become an important complementary and alternative treatment for chronic pain and is now a well-established option in the clinical management of chronic neuropathic pain (9). It shows considerable potential for relieving pain, improving limb function, and enhancing quality of life. CT involves applying cups (typically made of glass, clay, or similar materials) to the skin (10, 11). The cups are attached using mechanical suction or negative pressure generated by heat, which promotes local blood circulation (12, 13). CT is primarily categorized into two forms: dry cupping therapy (DCT) and wet cupping therapy (WCT). DCT relies solely on the suction force of the cup applied to the skin to produce its therapeutic effects. WCT involves making small incisions on the patient’s skin to allow blood and serum to flow out, thereby enhancing the efficacy observed with DCT (13, 14). Research into the mechanisms of CT is ongoing. Its main therapeutic effects are believed to arise from improving local blood circulation, reducing inflammation, and facilitating the clearance of pain-mediating substances (15, 16).

**Figure 1:**
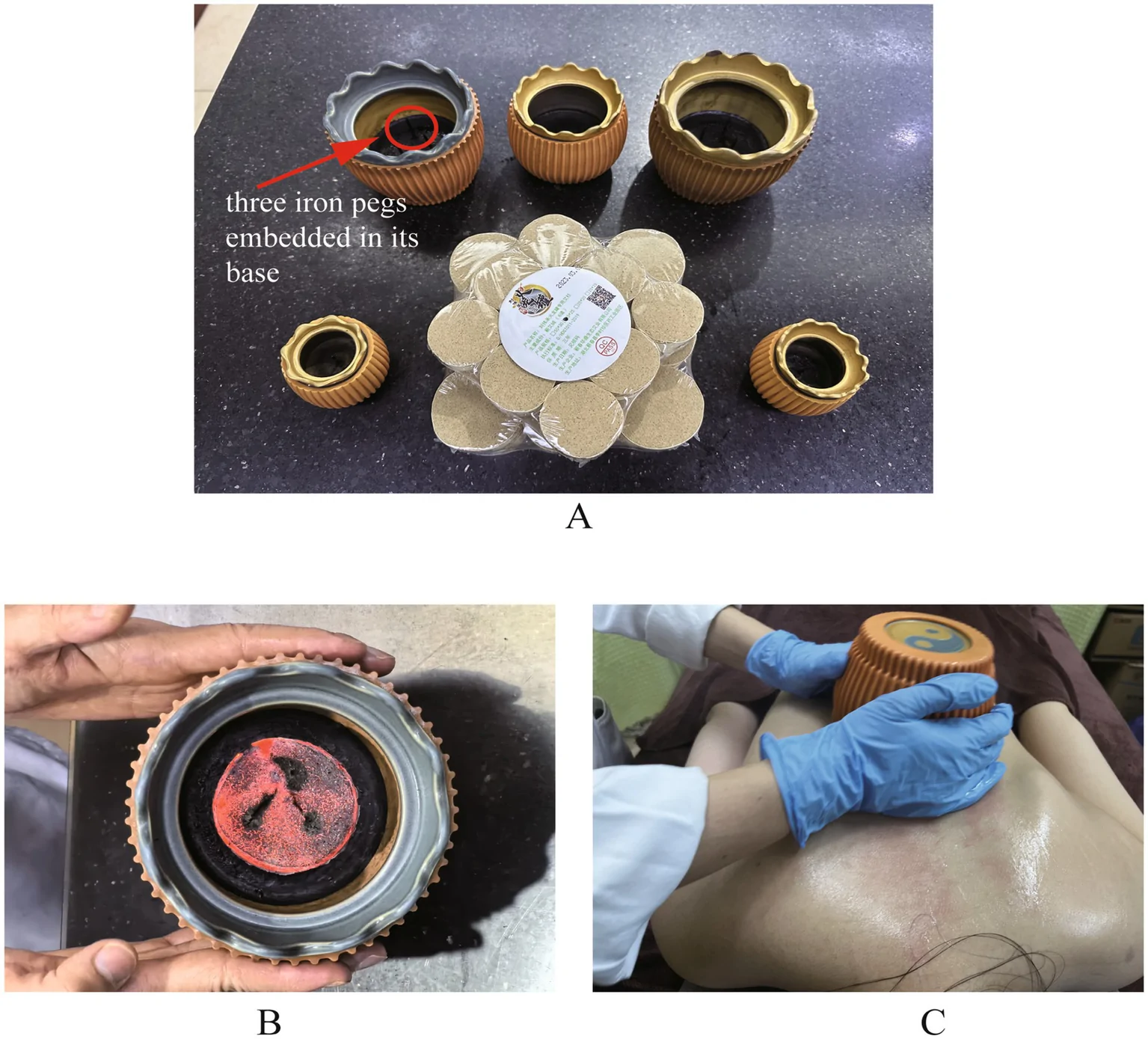
**(A)** Three iron pegs embedded in its base. **(B)** Practitioners hold the FDC (moxibustion is burning). **(C)** The practitioner apply specialized movements to the skin.

FDC therapy represents a further innovation in CT. Unlike conventional WCT and DCT, the FDC is a wide-mouthed clay vessel with three iron pegs embedded in its base (Figure 1A). Practitioners attach moxa sticks to these internal pegs and ignite them. The burning moxa allows the FDC to deliver both the heat-induced negative pressure of cupping and the therapeutic benefits of moxibustion. Furthermore, the rim of the cup has a regular, wavy contour (Figure 1B). This uneven edge enables the cup to rotate and slide simultaneously along the skin surface, a combined motion that effectively stimulates the patient’s acupoints (17, 18).

The FDC also features a unique manipulation technique. The practitioner systematically rotates and slides the cup along the patient’s meridians (Figure 1C), an approach that offers an innovative variation on standard moxibustion therapy. To perform the treatment, the practitioner holds the cup with both hands and uses the hypothenar eminence of the dominant hand to push the device, guiding it along the meridians. At the same time, both hands oscillate the vessel to create a clockwise or counterclockwise rotation. The practitioner monitors the local area to determine the appropriate moxibustion dose. Adequate treatment at a specific site may result in skin redness, pore dilation, or a sensation of heat reported by the patient. Once these signs appear, the cup is moved to the next target area. The choice of treatment sites and the overall treatment duration depend on the patient’s specific condition. This distinct handling method also ensures that the moxa burns completely.

FDC therapy is an innovative TCM therapy endorsed by the National Administration of Traditional Chinese Medicine. Its clinical application is growing and demonstrates positive therapeutic outcomes. There is currently no synthesized evidence evaluating the specific impact of FDC therapy on SHS. This absence of data restricts evidence-based support for its clinical use. Consequently, this review aims to summarize and assess the clinical evidence for FDC therapy in the treatment of SHS. The objective is to provide a reliable evidence-based reference for clinical practice.

## 2 Materials and methods

The protocol for this review was registered with PROSPERO in December 2025 (CRD420251249825). The detailed protocol is available at https://www.crd.york.ac.uk/PROSPERO/view/CRD420251249825. This review strictly adheres to the PRISMA 2020 statement and the PRISMA checklist requirements.

### 2.1 Literature search

This review searched four Chinese databases, including CNKI, Wanfang, VIP, and SinoMed, and 7 English databases, including PubMed, Embase, Web of Science, CINAHL, Ovid, Scopus, and the Cochrane Library. The search period was set from the inception of each database to 12 December 2025, with no language restrictions. All search strategies are provided in Supplementary material 1.

### 2.2 Inclusion criteria

We follow the PICOS to establish the inclusion criteria for the literature:

*Participants (P)*: Patients with a clinically confirmed diagnosis of SHS following ischemic or hemorrhagic stroke, verified by CT or MRI. We do not restrict age, gender, disease duration, or the stage of SHS (Stage I–III).

*Interventions (I)*: The experimental group received FDC therapy. It could be administered either as a monotherapy or as an adjunctive therapy combined with other treatments.

*Comparators (C)*: The control group received routine rehabilitation, conventional nursing care, or other treatments without FDC.

*Outcomes (O)*:

(1) *Upper motor function*: Using the Fugl-Meyer Assessment for Upper Extremity (FMA-UE)/ Fugl-Meyer Assessment (FMA) to assess.
(2) *Pain intensity*: Using the Visual Analog Scale (VAS) or Numerical Rating Scale (NRS) to assess.
(3) *Clinical efficacy*:

①*Cured*: Pain in the affected shoulder and hand disappears, local swelling subsides, muscles show no atrophy, and joint mobility reaches the normal range;
②*Significant improvement*: Pain in the affected shoulder and hand is reduced, local swelling alleviates, muscle atrophy is relieved, and joint mobility increases;
③*Improvement*: Pain in the affected shoulder and hand and local swelling are slightly reduced, muscle atrophy shows slight improvement, and joint mobility slightly improves;
④*Ineffective*: No significant change in pain, swelling, joint mobility, or muscle atrophy in the affected shoulder and hand, or symptoms may gradually worsen.

(4) *Hand Swelling*: Water Displacement Method/Circumference Measurement.

*Study Design (S)*: Randomized Controlled Trials (RCTs).

### 2.3 Exclusion criteria

Non-RCT study designs; Duplicate publications; Full-text inaccessibility.

### 2.4 Data extraction and management

We used EndNote21 for literature management and screening. Two reviewers (YX and HS) screened and excluded literature in accordance with the inclusion and exclusion criteria. Simultaneously, a literature characteristics table was used to independently extract the basic characteristics of literature meeting the inclusion criteria, including: Sample Size, Age and Gender distribution, Stroke Type, First-Ever Onset Status, Duration after Stroke Onset, Grading of SHS, Unilateral/Bilateral SHS, Specifics of the Intervention and Comparator, Treatment Details, Duration, Meridian-Following Status, Whether the control group intervention consisted solely of Rehab. Any divergences were resolved in a discussion among (YX, HS, and YY).

### 2.5 Risk of Bias assessment

Two reviewers (YX and HS) independently used the ROB2 (19) to assess the risk of bias. Based on Risk of Bias guidelines we evaluated each signal questions across five domains, the risk of bias for each study was classified as low risk, some concerns, or high risk, and visual representations of the results were generated.

### 2.6 Quality of evidence assessment

We used the GRADEpro GDT online tool to assess and recommend the quality of each outcome evidence (20). The initial quality grade of an RCT is high, but it may be downgraded due to factors such as risk of bias, inconsistency, indirectness, imprecision, and publication bias. The final evidence will be categorized into: High, Moderate, Low, and Very Low. We constructed a detailed SoF table to present specific details regarding the quality of evidence.

### 2.7 Statistical analysis

All statistical analyses, including standard meta-analyses, sensitivity analyses, subgroup analyses, and meta-regression, were conducted utilizing the *meta* (version 8.2–1) and *metafor* (version 4.8–0) packages in R. To ensure methodological transparency, a comprehensive list of the utilized R packages and their specific versions is provided in Supplementary material 1. Trial Sequential Analysis (TSA) was executed using TSAViewer software (version 0.9.5.10 Beta).

This review expressed binary outcomes as Risk Ratios (RR) and continuous outcomes as Standardized Mean Differences (SMD). We calculated 95% confidence intervals and prediction intervals for all outcomes. Considering the diversity of clinical operation details of FDC therapy and the anticipated clinical and methodological diversity across the included studies, we adopted the random-effects model as the primary analytic approach for all analyses in this study. For the clinical efficacy outcome, data categorization was standardized to facilitate effect size pooling and clinical interpretation. Specifically, patient cases reported as cured, markedly effective, or effective were merged into a unified effective classification to compute the overall effective rate across groups.

If high heterogeneity is present (I^2^ > 50%), to explore the potential sources of heterogeneity, we will conduct the pre-specified exploratory following subgroup analyses based on the clinical characteristics of SHS and operational features of FDC. Seven of the 11 trials did not report SHS stage. Because the extent of missing data precluded a meaningful subgroup analysis by stage, all trials were pooled irrespective of stage in the primary analysis.

(1) Unilateral/Bilateral SHS;
(2) Duration after stroke onset;
(3) First-ever onset status;
(4) Session Duration;
(5) Treatment frequency;
(6) Total intervention duration;
(7) Meridian-following status;
(8) Sample size;
(9) Whether the control group intervention consisted solely of rehab.

We used the leave-one-out method for sensitivity analysis to assess the robustness of the results. Moreover, to ensure that the study findings were not affected by publication bias, when a particular outcome included ≥10 studies, we also used funnel plots, Egger’s test, and the trim-and-fill method to evaluate the presence of publication bias. For outcomes encompassing more than 10 studies that exhibited high heterogeneity, a univariate meta-regression was further executed utilizing the publication year as a continuous covariate. To mitigate the risk of type I and type II errors arising from sparse data and repetitive significance testing, TSA was conducted for the primary outcomes of clinical efficacy. Establishing the type I error risk (*α*) at 5% and the type II error risk (*β*) at 20%. The pooled evidence was deemed robust and conclusive if the cumulative Z-curve successfully crossed the trial sequential monitoring boundary or surpassed the required information size (RIS) threshold.

## 3 Results

### 3.1 Literature search results

**Figure 2:**
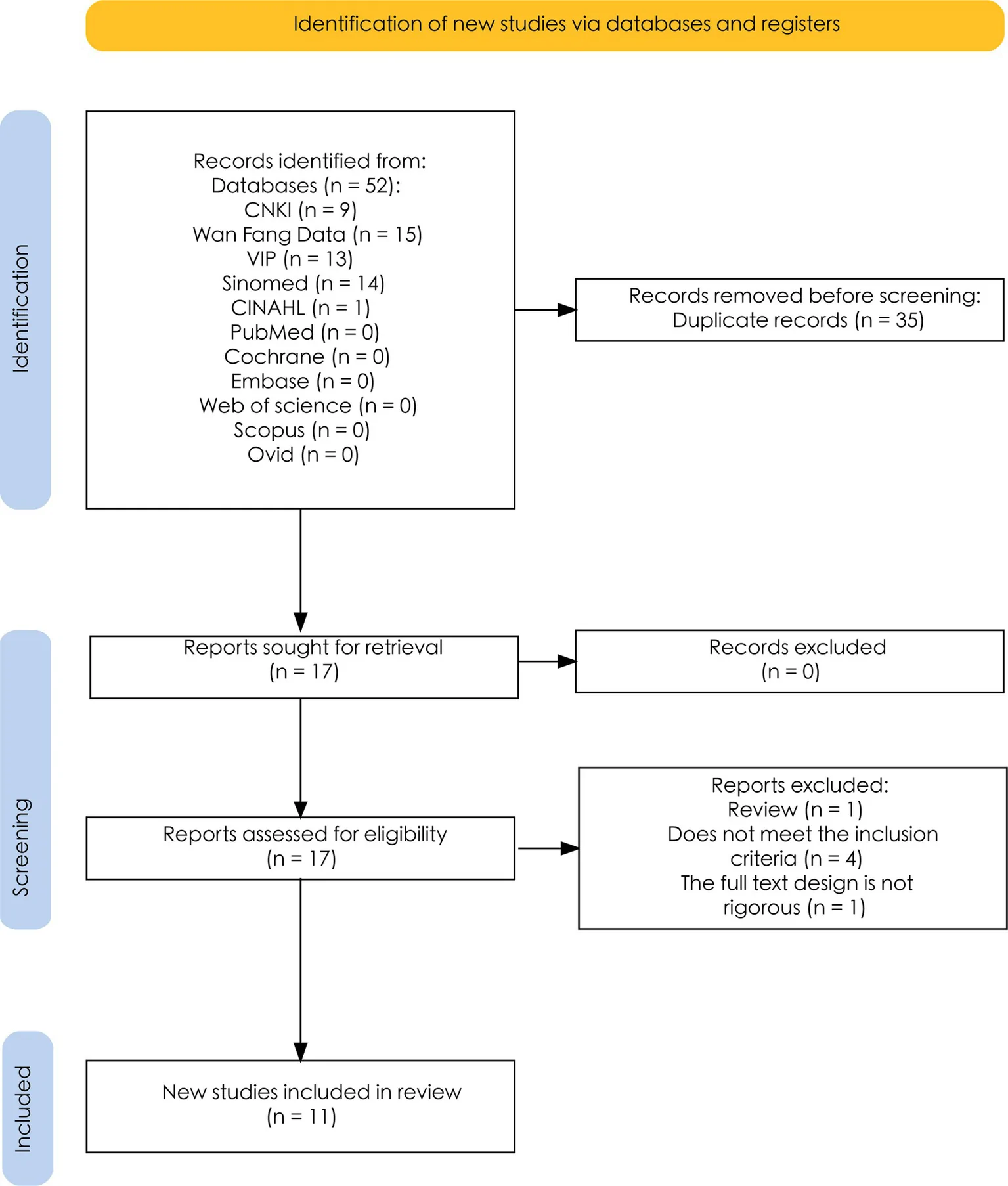
PRISMA flow diagram.

After searching in 11 databases, we obtained a total of 52 documents (1 from CINAHL, 51 from Chinese databases). After manually removing duplicates, reviewing abstracts, and reading full texts, we identified 11 RCTs for inclusion. Notably, FDC therapy, as an innovative TCM therapy, all included studies were retrieved exclusively from Chinese databases. According to the PRISMA 2020 statement, we provide a detailed literature selection process in the PRISMA Flow Diagram in Figure 2.

### 3.2 Characteristics of included studies

A total of 11 RCTs (21–31) published during 2022–2025 comprised 1,087 patients. Eight RCTs are SHS following ischemic stroke, and one RCT is SHS following ischemic stroke and hemorrhagic stroke, while two studies did not specify the stroke type.

The severity of SHS was primarily classified as Stage I (*n* = 3) or Stage I–II (*n* = 1), with the 7 RCTs not reporting. Regarding the laterality of SHS, 5 RCTs involved unilateral SHS, 1 RCT involved bilateral SHS, 2 RCTs included Unilateral and Bilateral SHS, and 3 RCTs did not specify. The detailed characteristics of the included studies are summarized in Table 1.

**Table 1:** Characteristics of studies.

Study	Sample size (Exp/Com)	Stroke type	Grading of SHS	Unilateral/bilateral SHS	Duration after stroke onset
Liu et al. (22)	71 (36/35)	Ischemic Stroke	1	U + B	1–3 M
Fan et al. (21)	62 (31/31)	Ischemic Stroke	NS	U	12 M
Xu et al. (24)	66 (33/33)	Ischemic Stroke	1	NS	NS
Wei et al. (23)	76 (38/38)	Ischemic Stroke and Hemorrhagic Stroke	NS	U + B	≤3 M
Ren et al. (26)	52 (26/26)	Ischemic Stroke	NS	U	NS
Wu et al. (27)	84 (42/42)	Ischemic Stroke	NS	U	1–3 M
Yuan et al. (28)	122 (61/61)	Ischemic Stroke	1	NS	NS
Deng et al. (25)	116 (58/58)	NS	NS	B	NS
Guo et al. (30)	120 (60/60)	Ischemic Stroke	1–2	U	2 W–3 M
Chen et al. (29)	200 (100/100)	NS	NS	NS	NS
Lei et al. (31)	118 (59/59)	Ischemic Stroke	NS	U	6 M

First-ever onset status	Treatment details; duration	Meridian-following status	Intervention
YES+NO	30–40 min, once/2 day; 21d	Yes	Rehab+FDC
YES	25 min, once/day; 14d	No	Com + FDC
NS	30 min, once/day, 5 day/w;28d	Yes	Com + FDC
YES	20 min, once/day, 5d/w; 28d	Yes	Com + FDC
NS	20 min, once/day, 5d/w; 30d	Yes	Com + FDC
NS	25 min, once/day; 28d	No	Com + FDC
YES	30–40 min, once/Every Other Day; 24d	Yes	Com + FDC
YES	30 min, once/day, 5 day/w; 28d	Yes	Rehab+FDC
NS	20 min, 5 day/w; 30d	Yes	Com + FDC
NS	20 min, 5 day/w; 28d	Yes	Com + FDC
YES	20 min, once/day; 52d	No	Com + FDC

Comparator	Gender (man/woman)	Age (exp/com)
Rehab+Moxibustion	exp (23/13); com (24/11)	exp (60.15 ± 7.49); com (61.43 ± 6.27)
Nedding of xingnaokaiqiao+Rehab	exp (23/8); com (24/7)	exp (64.73 ± 7.40); com (65.31 ± 8.62)
Rehab	exp (22/11);com(26/7)	exp (63.09 ± 8.92); com (64.27 ± 9.36)
Rehab	exp (23/15);com(21/17)	exp (65.12 ± 4.85); com (64.23 ± 4.34)
Rehab	exp (17/9);com(15/11)	exp (50.37 ± 4.63); com (50.13 ± 4.37)
Warm acupuncture moxibustion	exp (24/18);com(25/17)	exp (62.98 ± 7.74); com (62.85 ± 7.63)
Refined rehabilitation	exp (33/28);com(36/25)	exp (55.27 ± 8.03); com (55.36 ± 8.15)
Intramuscular taping	exp (35/23);com(36/22)	exp (56.39 ± 6.29); com (57.14 ± 7.13)
Rehab	exp (34/26);com(28/32)	exp (56.40 ± 4.20); com (55.90 ± 4.10)
Rehab	exp (50/50);com(51/49)	exp (45.32 ± 10.21); com (46.11 ± 9.89)
Nedding of xingnaokaiqiao+Rehab	exp (32/27);com(30/29)	exp (58.17 ± 6.98); com (59.37 ± 6.16)

Order of operations	Key targeted acupoints	Duration of condition
GV-BL-TYMH(Affected)	GV14, BL12, GB21, LI15, Extra Point, SI9, LI11, TE4, LI4, Ashi Point	NS
From Shoulder to Hand–From Hand to Shoulder	NS	exp (26.45 ± 5.27)d; com(26.77 ± 6.32)d
TYMH	LI15, LI14, LI11, LI4	exp (36.86 ± 9.07)d; com(35.3 ± 6.72)d
GV-LI-TE	LI11, LI4, LI15, TE3, TE4	exp (2.01 ± 0.08)m; com(1.42 ± 0.54)m
GV-LI-TE	LI11, LI4, LI15, TE3, TE4	NS
From Hand to Shoulder	NS	exp (26.87 ± 5.42)d; com(26.71 ± 5.39)d
GV-LI-TE-Scraping Acupoints-GV-LI-TE	LI11, LI4, LI15, TE3, TE4	exp(15.31 ± 2.47)d;com(15.50 ± 2.38)d
GV-TYMH(Affected)	GV14, BL12, GB21, LI11, TE4, Ashi Point	NS
GV-LI-TE	LI11, LI4, LI15, TE3, TE4	exp (42.3 ± 3.6)d;com (43.5 ± 4.1)d
LI-PC- TE	LI15, LI11, LI4, TE5, PC6	exp (3.8 ± 1.6)d; com (4.1 ± 1.5)d
From Shoulder to Hand	NS	exp (5.87 ± 1.24)w; com (6.02 ± 1.15)w

### 3.3 Risk of Bias assessment

In the domain of Randomization Process, the study by Liu et al. (22) was rated as High Risk due to the use of an admission-order-based grouping method. Only Lei et al. (31) was rated as Low Risk and 9 RCTs were rated as Some Concerns because they lacked a specific description regarding allocation concealment.

**Figure 3:**
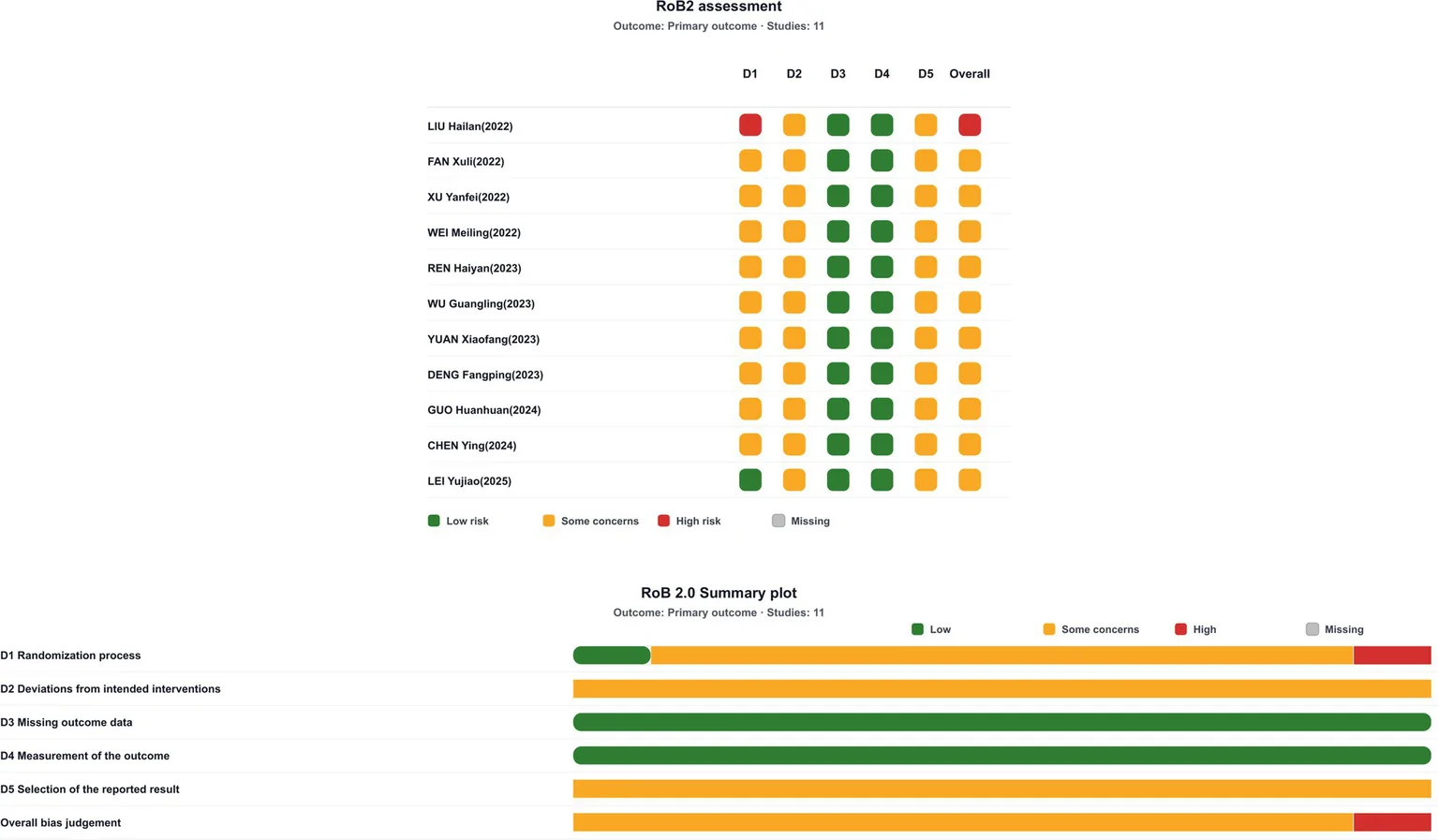
Risk of bias assessment.

In the domain of Deviations from Intended Interventions and Selection of the Reported, all 11 RCTs were rated as Some Concerns. In the domain of Missing Outcome Data and Measurement, all 11 RCTs were rated as Low Risk. Details in Figure 3.

### 3.4 Major outcome

#### 3.4.1 Upper motor function

**Figure 4:**
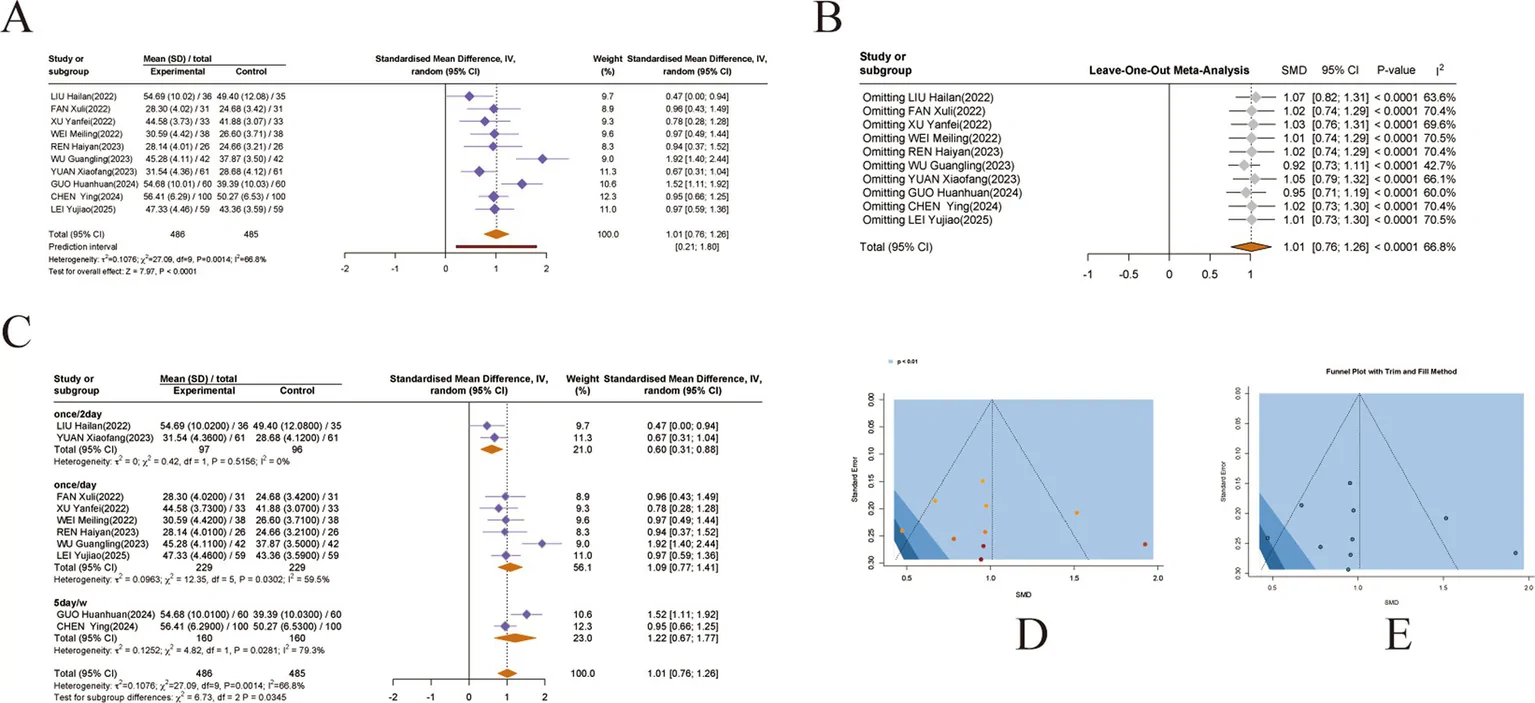
**(A)** Summary statistics of upper motor function. **(B)** Sensitivity analysis of upper motor function. **(C)** Treatment frequency of upper motor function. **(D)** Funnel plot of upper motor function. **(E)** The trim-and-fill of upper motor function.

In terms of upper motor function, 10 RCTs (*n* = 971) demonstrated that FDC therapy can improve upper motor function (SMD = 1.01, 95% CI: [0.76 to 1.26], *p* < 0.0001) (Figure 4A), and the prediction interval [0.21, 1.80] did not cross zero. Even if moderate heterogeneity was found (*I*^2^ = 66.8%), sensitivity analysis identified that Wu et al. (27) was the source of heterogeneity, and after excluding Wu et al. (27) I^2^ decreased to 42.7% (SMD = 0.92, *p* < 0.0001) (Figure 4B). To explore the source of this heterogeneity, exploratory subgroup analysis was conducted. The subgroups of Treatment Frequency (Figure 4C) exhibited significant statistical differences. It is noteworthy that once/2 day showed no statistical heterogeneity (*I*^2^ = 0). The funnel plot appeared symmetrical (Figure 4D), Egger’s test confirmed no significant small-study effects (*t* = 0.44, *p* = 0.6683), and the trim-and-fill analysis imputed zero missing studies (Figure 4E). There is no evidence of publication bias for upper motor function. Furthermore, confirmed that publication year was not a significant covariate contributing to the heterogeneity (*p* = 0.411, *R*^2^ = 0) (Supplementary material 1). Based on the GRADE criteria, the certainty of evidence was assessed as Low; the rating was downgraded due to the serious risk of bias in the lack of blinding and serious indirectness (all included patients were Chinese, limiting generalizability to other populations, and 6 RCTs did not report SHS stage, precluding assessment of effect modification by disease severity) (Supplementary material 1).

#### 3.4.2 Clinical efficacy

**Figure 5:**
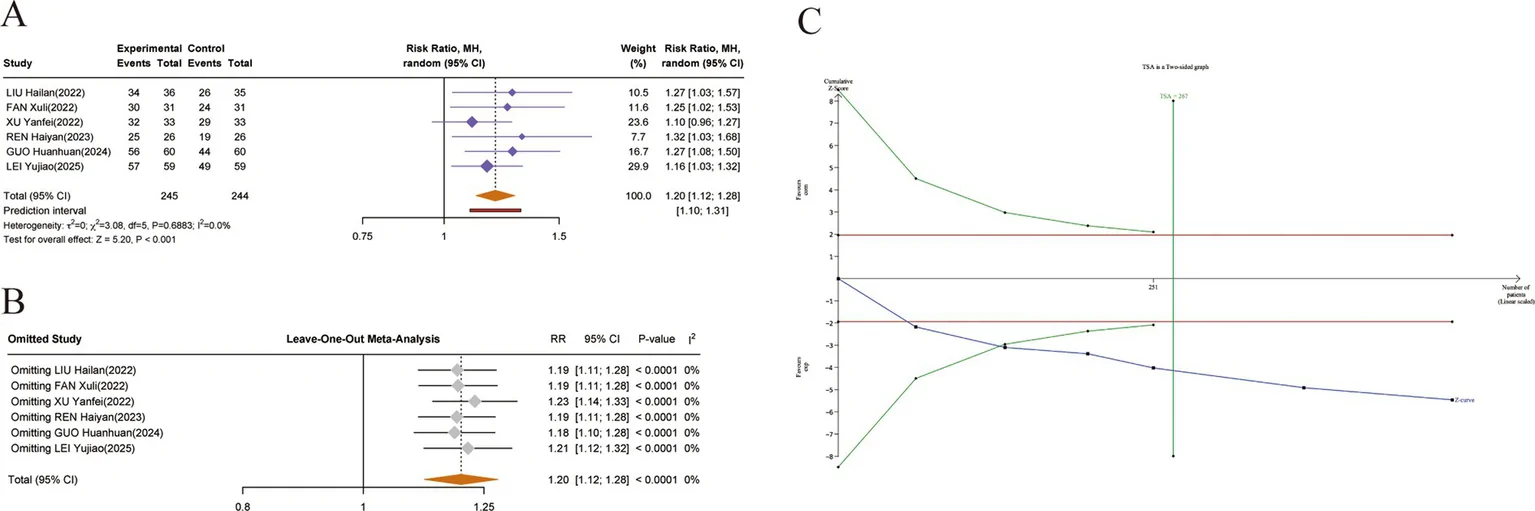
**(A)** Summary statistics of clinical efficacy. **(B)** Sensitivity analysis of clinical efficacy. **(C)** The result of TSA.

In terms of clinical efficacy, six RCTs (*n* = 489) (Figure 5A) demonstrated that FDC therapy shows a trend that is beneficial for improving the clinical efficacy of patients with SHS (RR = 1.20, 95% CI:[1.12 to 1.28], *p* < 0.0001), and no heterogeneity was observed (I^2^ = 0%), and the prediction interval [1.10–1.31] did not cross 1. Sensitivity analysis also demonstrates the homogeneity among the research results (Figure 5B). Furthermore, TSA revealed that the RIS was 267 (Figure 5C), and the accumulated sample size (*n* = 489) has exceeded this threshold. Although the TSA suggests that RIS has now reached the conclusive criteria and future studies are unlikely to change the direction of the existing conclusions, this does not enhance the certainty of the evidence for this subjective composite outcome. As the outcome assessment relies on subjective judgment and 3 RCTs did not report SHS stage, precluding assessment of effect modification by disease severity, the GRADE is Low.

### 3.5 Secondary outcome

#### 3.5.1 Hand swelling

**Figure 6:**
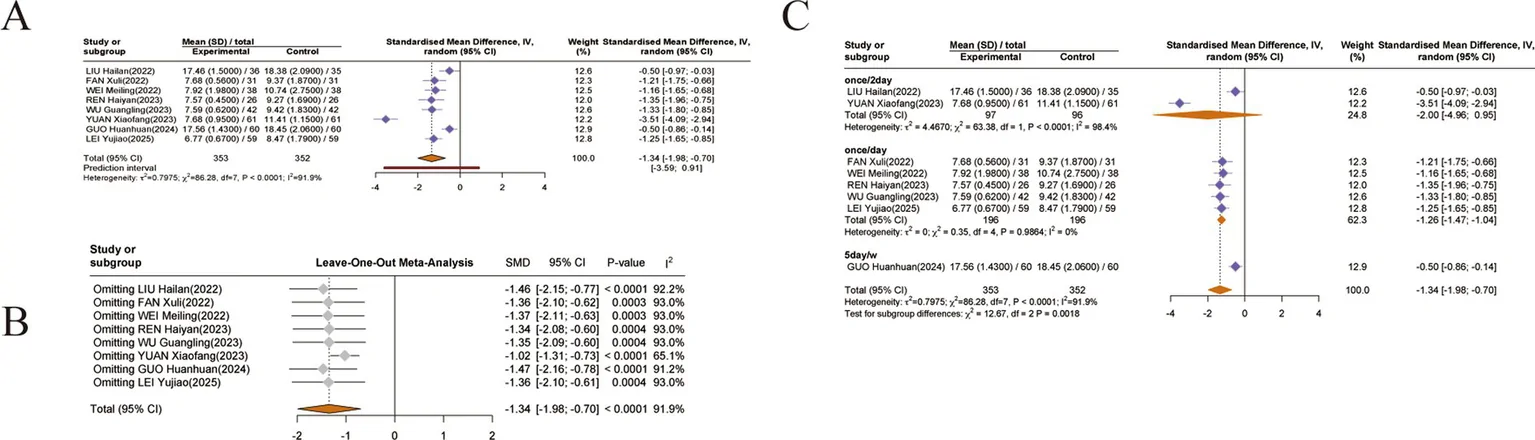
**(A)** Summary statistics of hand swelling. **(B)** Sensitivity analysis of hand swelling. **(C)** Treatment frequency of hand swelling.

In terms of Hand Swelling, 8 RCTs (*n* = 705) demonstrated that the FDC therapy shows a trend that is beneficial for improving the Hand Swelling in patients with SHS (SMD = −1.34, 95% CI:[−1.98 to −0.70], *p* < 0.0001), but there was significant heterogeneity (I^2^ = 91.9%) (Figure 6A), and the prediction interval [−3.59–0.91] did cross zero. Sensitivity analysis showed that if Yuan et al. (28) was excluded, the heterogeneity decreased to 65.1%, while the overall effect size remained significant (SMD = −1.02, 95% CI:[−1.31 to −0.73], *p* < 0.0001) (Figure 6B). To explore the source of this heterogeneity, exploratory subgroup analysis was conducted. Subgroup analysis based on treatment frequency demonstrated complete homogeneity (I^2^ = 0) within the once/day subgroup (Figure 6C). According to the GRADE framework, the overall certainty of evidence for this outcome was assessed as Very Low. The evidence was downgraded by one level for risk of bias due to the inherent difficulty in blinding. It was additionally downgraded by one level for indirectness, as 5 RCTs s did not report SHS stage, limiting the assessment of effect modification by disease severity, and all patients were Chinese. Crucially, the evidence was downgraded by two further levels for very serious inconsistency, evidenced by the exceptionally high heterogeneity (I^2^ = 91.9%) and a wide prediction interval that crosses zero.

#### 3.5.2 Pain intensity

**Figure 7:**
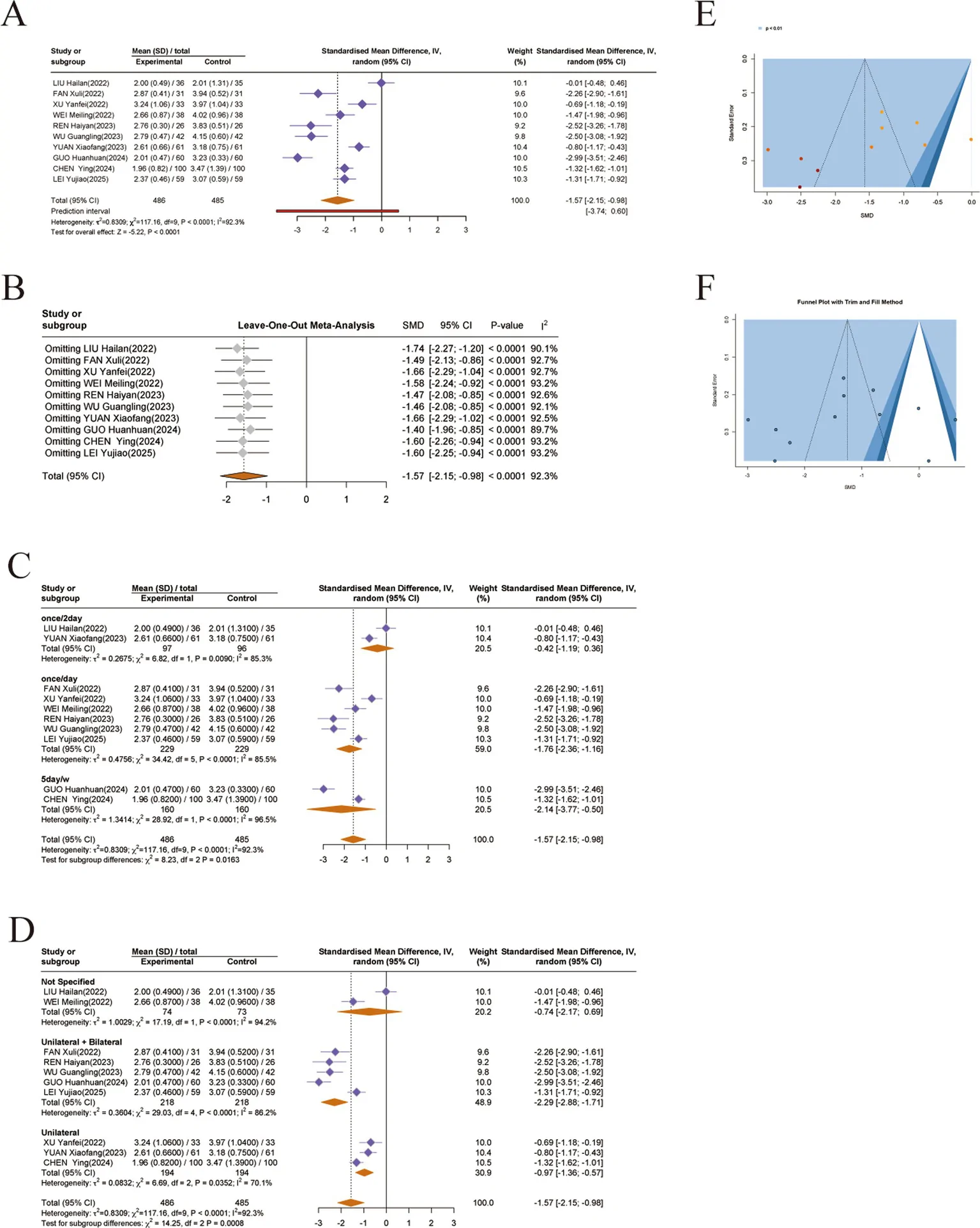
**(A)** Summary statistics of pain intensity. **(B)** Sensitivity analysis of pain intensity. **(C)** Treatment frequency of pain intensity. **(D)** Unilateral/bilateral SHS of pain intensity. **(E)** Funnel plot of pain intensity. **(F)** The trim-and-fill of pain intensity.

The pooled results from 10 studies indicate that FDC therapy shows a trend that is beneficial for improving pain intensity in patients with SHS (SMD = −1.57, 95% CI:[−2.15 to −0.98], *p* < 0.0001), but there was significant heterogeneity (I^2^ = 92.3%), and the prediction interval [−3.74–0.60] did cross zero. (Figure 7A). Given the severity of this heterogeneity, this pooled SMD is presented solely for completeness of statistical reporting; it should not be interpreted as a single summary effect. Although Liu et al. (22) appear to cross the no-effect line when observed in the forest plot, sensitivity analysis reveals that even if Liu et al. (22) is excluded, the research remains robust and maintains a high effect size (SMD = −1.74, 95% CI: [−2.27 to −1.20], *p* < 0.0001) (Figure 7B). Based on the pre-specified exploratory subgroup analysis, it was found that Treatment Frequency (*p* = 0.0163) (Figure 7C) and Unilateral/Bilateral SHS (*p* = 0.0008) (Figure 7D) were meaningful subgroups, while the remaining subgroups showed no statistical differences (*p* > 0.05 for all) (Supplementary material 1).

We observed a slight asymmetry in the funnel plot (Figure 7E), with a *p*-value of 0.1526 (*t* = −1.58) obtained from Egger’s test. Most notably, the trim-and-fill method identified 2 potentially missing studies. After incorporating these studies, the SMD was −1.25 (95% CI:[−1.90 to −0.60], *p* = 0.0002) (Figure 7F), indicating that the analysis suggested a possible mild bias, but the adjusted results remained robust.

The certainty of evidence for pain intensity was assessed as very low. The evidence was downgraded by one level for risk of bias due to the inherent difficulty in blinding. It was additionally downgraded by one level for indirectness, because six RCTs did not report the SHS stage and all patients were Chinese. Furthermore, it was downgraded by two additional levels for very serious inconsistency, as evidenced by high statistical heterogeneity (I^2^ = 92.3%) and a wide prediction interval that crosses the no-effect line.

However, this pooled estimate must be interpreted with extreme caution, if at all, due to prohibitively high statistical heterogeneity (I^2^ = 92.3%) and a 95% prediction interval that widely crosses the line of no effect ([−3.74, 0.60]). The high level of inconsistency suggests that the observed effect is not stable across studies, and the single pooled SMD may not represent a clinically meaningful summary. Therefore, we present this result as a trend only.

## 4 Discussion

This review synthesized 11 RCTs (*n* = 1,087). We aimed to evaluate the utility of FDC therapy for post-stroke SHS. Our pooled data suggest that FDC therapy may offer potential clinical benefits. Specifically, the FDC therapy showed a positive trend toward improving upper motor function (SMD = 1.01, 95% CI:[0.76 to 1.26], *p* < 0.0001) and clinical efficacy (RR = 1.20, 95% CI:[1.12 to 1.28], *p* < 0.0001). It also demonstrated a potential ability to reduce hand swelling (SMD = −1.34, 95% CI:[−1.98 to −0.70], *p* < 0.0001) and alleviate pain intensity (SMD = −1.57, 95% CI:[−2.15 to −0.98], *p* < 0.0001).

Moderate heterogeneity (I^2^ = 66.8%) was observed in the pooled results for upper motor function. Sensitivity analysis revealed that excluding the study by Wu et al. (27) reduced the heterogeneity I^2^ to 42.7%, while the overall effect remained robust. Notably, the control group in Wu et al. (27) received warm acupuncture, whereas the experimental group received warm acupuncture combined with FDC. This trial yielded a substantially higher effect size (SMD = 0.92). This suggests a potential synergistic effect when combining FDC therapy with warm acupuncture to improve upper motor function.

Furthermore, the prediction interval indicates that FDC therapy is likely to maintain this clinical advantage in future practice. Although the funnel plot and trim-and-fill analysis indicated no publication bias, these findings require cautious interpretation, because all included studies originated from China. Since all the patients included in this study were Chinese and the operators were trained traditional Chinese medicine nurses, the study’s extrapolation was limited.

In the term of clinical efficacy, the pooled results demonstrated zero heterogeneity (I^2^ = 0%). Both the sensitivity analysis and the prediction interval support the potential advantage of FDC therapy. TSA calculated an RIS of 267 patients. Our actual pooled sample size reached 489. This confirms that future homogeneous studies are unlikely to alter the positive direction of this conclusion. However, the exclusive reliance on Chinese literature may exaggerate the effect size. Additionally, this outcome partially relies on the subjective judgment of researchers. Therefore, these results serve as indicative, rather than definitive, evidence of comprehensive efficacy. Future trials must utilize standardized, objective, and reproducible outcome measures to enhance clinical interpretability.

The pooled results for hand swelling exhibited high heterogeneity (I^2^ = 91.9%). Sensitivity analysis showed that excluding the study by Yuan et al. (28) decreased the I^2^ to 65.1%. Yuan et al. (28) reported a large sample size (*n* = 122) and compared refined nursing with FDC plus refined nursing, which partially explained the variance. Pre-specified subgroup analyses revealed complete homogeneity (I^2^ = 0%) within the once/day treatment frequency group. This suggests that frequent FDC therapy interventions may be necessary to reduce hand swelling effectively. Other subgroup analyses and meta-regression failed to identify further sources of heterogeneity. The inherent difficulty of implementing blinding in TCM external therapies likely contributes to the inter-study variance. Crucially, the 95% prediction interval crossed the no-effect line. This indicates that future studies could alter the current findings. Consequently, we cannot definitively conclude that FDC therapy effectively resolves hand swelling; the pooled SMD merely highlights a positive trend. Validating this trend requires future studies with standardized operational protocols and higher evidence certainty.

The pooled data reveal a positive trend for FDC therapy in alleviating pain intensity (SMD = −1.57). However, this analgesic effect must be interpreted with extreme caution due to severe heterogeneity and a prediction interval [−3.74 to 0.60] that explicitly crosses the no-effect line. Exploratory subgroup analyses found treatment frequency and SHS laterality as potential sources of this variance. However, we explicitly acknowledge that these subgroup findings are strictly hypothesis-generating, rather than confirmatory. The observed variations across different treatment frequencies cannot be interpreted as a definitive dose–response relationship, as they could merely reflect other confounding study-level characteristics, such as different patient populations, varying baseline SHS stages, or the highly heterogeneous comparators across the included trials. Therefore, these subgroup results only serve to generate preliminary clinical hypotheses for future investigation. Importantly, FDC therapy is a highly operator-dependent external therapy. Variations in cupping manipulation techniques, applied pressure, and overall practitioner proficiency directly impact intervention quality, likely driving the massive inter-study heterogeneity.

Furthermore, we observed that the study by Liu et al. (22), which allocated patients based on admission order, crossed the line of no effect and yielded results completely inconsistent with the other included trials. This methodological deviation may account for the observed heterogeneity. Furthermore, pain is a purely subjective endpoint. Pain scores are heavily confounded by patient expectations, cultural backgrounds, and the specific measurement scales utilized. The physical characteristics of FDC make blinding impossible for both patients and practitioners, which systematically amplifies expectation bias and further explains the high heterogeneity.

Methodologically, sensitivity analysis confirmed that excluding the outlier study by Liu et al. (22) preserved a robust effect size (SMD = −1.74). Yet, trim-and-fill analysis detected two potentially missing studies. Imputing these trials lowered the adjusted effect size to −1.25, confirming the presence of mild publication bias. Importantly, this adjusted estimate remained highly statistically significant (*p* = 0.0002). This demonstrates that although publication bias artificially inflated the effect magnitude, the underlying trend toward pain relief is genuine. Nevertheless, constrained by the lack of blinding and severe heterogeneity, the overall GRADE certainty for this outcome was assessed as very low. Given the substantial heterogeneity observed in pain alleviation and a prediction interval that crosses the line of no effect, the overall effect size merely suggests a potential trend toward pain relief. Due to the very low certainty of evidence, these findings must be interpreted with extreme caution. We reiterate that due to I^2^ = 92.3% and a prediction interval crossing zero, the pooled SMD for pain intensity is reported only for transparency and should not be used as a basis for clinical inference. Future high-quality, rigorously blinded RCTs are imperative to provide definitive evidence regarding the analgesic benefits of FDC for patients with SHS. Moreover, there is a pressing need to develop standardized methodological protocols for FDC clinical trials. Such protocols must go beyond outlining basic treatment durations and workflows to detail the nuanced operational specifics of the intervention explicitly.

### 4.1 Potential mechanisms

FDC targets specific meridians and acupoints (17, 18). Our review identified LI11, LI4, LI15, TE3, and TE4 as the primary targets. LI11, LI4, and LI15 belong to the Large Intestine Meridian. Stimulating this meridian improves local blood circulation (32, 33). TE3 and TE4 belong to the Triple Energizer Meridian. Targeting these points helps reduce hand swelling (34, 35).

Current literature proposes several cellular mechanisms for these specific acupoints. General acupuncture research suggests that stimulating LI4 inhibits pro-apoptotic factors like c-Fos, caspase-3, and Bax. It may also upregulate the protective protein Bcl-2 (33, 35–37). Similarly, stimulating LI11 might promote the release of brain-derived neurotrophic factor (BDNF). This release supports neurogenesis and angiogenesis, which aids motor recovery (34–36, 38).

We must clearly separate these hypotheses from our evidence-based conclusions. The clinical trials in this review only evaluated clinical symptoms. They did not measure these biomolecular markers. Therefore, these neurogenic and anti-inflammatory pathways are extrapolated from related acupuncture research. They are not demonstrated mechanisms of FDC therapy. Future basic research must specifically investigate FDC to validate these molecular claims.

### 4.2 Strengths and limitations

This study presents several methodological strengths. It is the first systematic review and meta-analysis to synthesize the efficacy of FDC therapy for post-stroke SHS, strictly adhering to the PRISMA 2020 statement. We utilized robust methodological tools, including TSA, ROB-2, and the GRADE framework, to objectively evaluate the evidence. Furthermore, we systematically employed the leave-one-out sensitivity analysis and publication bias assessments (funnel plots, Egger’s test, and trim-and-fill method) to rigorously test the robustness of the pooled estimates against outlier studies and small-study effects.

Despite these strengths, several major limitations critically temper our conclusions:

Firstly, all 11 included RCTs originated exclusively from Chinese databases, with major international databases yielding zero results. This extraordinary reliance on a single-country evidence base makes extrapolation to non-Chinese healthcare settings highly limited.

Secondly, sensitivity analyses failed to resolve severe statistical heterogeneity for subjective outcomes. Although excluding Liu et al. (22) (which utilized high-risk admission-order randomization) confirmed a trend for pain intensity, the massive heterogeneity (I^2^ = 92.3%) remained unexplained. Similarly, for hand swelling, excluding Yuan et al. (28) only reduced the I^2^ to 65.1%, which is still substantial. The 95% prediction intervals for both outcomes critically crossed the line of no effect, indicating high uncertainty regarding FDC’s true efficacy in future varied settings.

Thirdly, pooling across heterogeneous comparators introduces significant clinical confounding. The control groups in the included trials varied widely, encompassing routine rehabilitation, refined nursing, intramuscular taping, and warm acupuncture. Conflating these fundamentally different clinical comparisons complicates the interpretation of FDC’s specific adjunctive benefit. While we pooled these data to evaluate the broad, overarching trend of FDC efficacy across diverse real-world neurorehabilitation settings, this methodological conflation means our pooled estimates must be interpreted with extreme caution. Future research must stratify by specific comparator types to answer more precise clinical questions.

Fourthly, methodological quality and the lack of Standardized Operating Procedures (SOPs) introduce substantial risk of bias. The inherent physical characteristics of FDC make blinding participants and personnel virtually impossible, amplifying performance and expectation biases. Moreover, none of the original RCTs provided pre-registered protocols. The current clinical practice of FDC lacks detailed SOPs regarding thermal intensity, specific manipulation techniques, and duration, which directly drives inter-study variance.

Lastly, the neurogenic and anti-inflammatory mechanisms discussed are extrapolated from related acupuncture literature.

In conclusion, given the Low to Very Low certainty of evidence across all outcomes, strong clinical recommendations cannot be justified at this stage. Future multi-center, adequately blinded RCTs adhering to CONSORT guidelines, employing clear SOPs, and stratifying by specific comparator types are imperative.

## Data Availability

The original contributions presented in the study are included in the article/Supplementary material, further inquiries can be directed to the corresponding author.
