# Correction: Effects of fire dragon cupping therapy for patients with post-stroke shoulder-hand syndrome: a systematic review and meta-analysis

**DOI:** 10.3389/fneur.2026.1965377

**Published:** 2026-08-25

**Authors:** Yiqiang Xu, Hongman Shen, Yue Yang

**Affiliations:** 1 Heilongjiang University of Chinese Medicine, Harbin, Heilongjiang, China; 2 First Affiliated Hospital, Heilongjiang University of Chinese Medicine, Harbin, Heilongjiang, China

**Keywords:** fire dragon cupping, reflex sympathetic dystrophy, shoulder-hand syndrome, stroke, traditional Chinese medicine

In the published article, **affiliation** Heilongjiang University of Chinese Medicine, Harbin, Heilongjiang, China was omitted from the paper. This affiliation has now been added for author(s) Yiqiang Xu, Hongman Shen.

Authors Yiqiang Xu and Hongman Shen were erroneously assigned to affiliation First Affiliated Hospital, Heilongjiang University of Chinese Medicine, Harbin, Heilongjiang, China

In addition, Author Hongman Shen was erroneously omitted as equal contributing author.

The original version of this article has been updated.

