# Supplementary material for: Effects of fire dragon cupping therapy for patients with post-stroke shoulder-hand syndrome: a systematic review and meta-analysis
Source: Front Neurol. 2026 Jul 30;17:1857245. doi: 10.3389/fneur.2026.1857245 (PMC13467949; doi:10.3389/fneur.2026.1857245)
Supplement: Supplementary file 1 [file Data_sheet_1.docx]

Supplementary Material

# 1 Search Strategy

## 1.1 Search strategy of PubMed

| Search number | Query | Results |
| --- | --- | --- |
| 1 | "Stroke"[Mesh] | 196113 |
| 2 | "Strokes" or "Cerebrovascular Accident" or "Cerebrovascular Accidents" or "Cerebral Stroke" or "Cerebral Strokes" or "Stroke, Cerebral" or "Strokes, Cerebral" or "Cerebrovascular Apoplexy" or "Apoplexy, Cerebrovascular" or "Brain Vascular Accident" or "Brain Vascular Accidents" or "Cerebrovascular Stroke" or "Cerebrovascular Strokes" or "Stroke, Cerebrovascular" or "Strokes, Cerebrovascular" or "Apoplexy" or "Stroke, Acute" or "Acute Stroke" or "Acute Strokes" or "Strokes, Acute" or "Cerebrovascular Accident, Acute" or "Acute Cerebrovascular Accident" or "Acute Cerebrovascular Accidents" or "Cerebrovascular Accidents, Acute" | 66795 |
| 3 | 1 OR 2 | 230712 |
| 4 | "Reflex Sympathetic Dystrophy"[Mesh] | 3690 |
| 5 | "Reflex Sympathetic Dystroph*" or "Dystrophy, Reflex Sympathetic" or "Sympathetic Dystrophy, Reflex" or "CRPS Type I" or "Type I, CRPS" or "Complex Regional Pain Syndrome, Type I" or "Sudek Atrophy" or "Atrophy, Sudek" or "Reflex Sympathetic Dystrophy Syndrome" or "Sympathetic Reflex Dystrophia" or "Syndrome, Reflex Sympathetic Dystrophy" or "Type I Complex Regional Pain Syndrome" or "Sudek's Atrophy" or "Atrophy, Sudek's" or "Sudeks Atrophy" or "Algodystrophic Syndrome" or "Algodystroph*" or "Shoulder-Hand Syndrome" or "Shoulder Hand Syndrome*" | 4716 |
| 6 | 4 OR 5 | 4716 |
| 7 | "Fire Dragon Cupping" or "Fire Dragon Pot" or "Fire Dragon Jar" or "Fire Dragon Can" or "Fire Dragon Pot Therapy" or "Huolong Pot" or "Huolong(Fire Dragon) Cupping" or "Huolong Guan" or "Huo Long Cupping" or "Huo Long Guan Therapy" or "Huo Long Cupping Therapy" or "Huo-Long Cupping Therapy" or "Huolong Guan Combined Therapy" or "Huolong Guan Nursing" or "Comprehensive Moxibustion With Huolong Cupping" or "Dragon Jar Comprehensive Moxibustion" or "Dragon Jar Comprehensive Moxibustion" or "Tcm Huolong Guan Therapy" | 32 |
| 8 | 3 AND 6 AND 7 | 0 |

## 1.2 Search strategy of Cochrane

| ID | Search | Hits |
| --- | --- | --- |
| #1 | MeSH descriptor: [Stroke] explode all trees | 18736 |
| #2 | (Strokes):ti,ab,kw OR (Stroke*, Acute):ti,ab,kw OR (Acute Stroke*):ti,ab,kw OR (Stroke*, Cerebral):ti,ab,kw OR (Stroke*, Cerebrovascular):ti,ab,kw | 24136 |
| #3 | (Cerebral Stroke*):ti,ab,kw OR (Cerebrovascular Stroke*):ti,ab,kw OR (Cerebrovascular Accident*):ti,ab,kw OR (Cerebrovascular Apoplexy):ti,ab,kw OR (Cerebrovascular Accident*, Acute):ti,ab,kw | 29525 |
| #4 | (Acute Cerebrovascular Accident*):ti,ab,kw OR (Apoplexy):ti,ab,kw OR (Apoplexy, Cerebrovascular):ti,ab,kw OR (Brain Vascular Accident*):ti,ab,kw | 5759 |
| #5 | #1 OR #2 OR #3 OR #4 | 52332 |
| #6 | MeSH descriptor: [Reflex Sympathetic Dystrophy] explode all trees | 295 |
| #7 | (Reflex Sympathetic Dystroph*):ti,ab,kw OR (Dystrophy, Reflex Sympathetic):ti,ab,kw OR (Sympathetic Dystrophy, Reflex):ti,ab,kw OR (CRPS Type I):ti,ab,kw OR (Type I, CRPS):ti,ab,kw | 482 |
| #8 | (Complex Regional Pain Syndrome, Type I):ti,ab,kw OR (Sudek Atrophy):ti,ab,kw OR (Atrophy, Sudek):ti,ab,kw OR (Reflex Sympathetic Dystrophy Syndrome):ti,ab,kw OR (Sympathetic Reflex Dystrophia):ti,ab,kw | 424 |
| #9 | (Syndrome, Reflex Sympathetic Dystrophy):ti,ab,kw OR (Type I Complex Regional Pain Syndrome):ti,ab,kw OR (Sudek's Atrophy):ti,ab,kw OR (Atrophy, Sudek's):ti,ab,kw OR (Sudeks Atrophy):ti,ab,kw | 423 |
| #10 | (Algodystrophic Syndrome):ti,ab,kw OR (Algodystroph*):ti,ab,kw OR (Shoulder-Hand Syndrome):ti,ab,kw OR (Shoulder Hand Syndrome*):ti,ab,kw | 741 |
| #11 | #6 OR #7 OR #8 OR #9 OR #10 | 1202 |
| #12 | (Fire Dragon Cupping):ti,ab,kw OR (Fire Dragon Pot):ti,ab,kw OR (Fire Dragon Jar):ti,ab,kw OR (Fire Dragon Can):ti,ab,kw OR (Fire Dragon Pot Therapy):ti,ab,kw | 19 |
| #13 | (Huolong Pot):ti,ab,kw OR (Huolong(Fire Dragon) Cupping):ti,ab,kw OR (Huolong Guan):ti,ab,kw OR (Huo Long Cupping):ti,ab,kw OR (Huo Long Guan Therapy):ti,ab,kw | 5 |
| #14 | (Huo Long Cupping Therapy):ti,ab,kw OR (Huo-Long Cupping Therapy):ti,ab,kw OR (Huolong Guan Combined Therapy):ti,ab,kw OR (Huolong Guan Nursing):ti,ab,kw OR (Comprehensive Moxibustion With Huolong Cupping):ti,ab,kw | 4 |
| #15 | (Dragon Jar Comprehensive Moxibustion):ti,ab,kw OR (Tcm Huolong Guan Therapy):ti,ab,kw | 2 |
| #16 | #12 OR #13 OR #14 OR #15 | 24 |
| #17 | #5 AND #11 AND #16 | 0 |

## 1.3 Search Strategy of Embase

| No. | Query | Results |
| --- | --- | --- |
| #1 | 'cerebrovascular accident'/exp | 532638 |
| #2 | 'accident, cerebrovascular':ab,ti OR 'acute cerebrovascular lesion':ab,ti OR 'acute focal cerebral vasculopathy':ab,ti OR 'acute stroke':ab,ti OR 'apoplectic stroke':ab,ti OR 'apoplexia':ab,ti OR 'apoplexy':ab,ti OR 'blood flow disturbance, brain':ab,ti OR 'brain accident':ab,ti OR 'brain attack':ab,ti OR 'brain blood flow disturbance':ab,ti OR 'brain insult':ab,ti OR 'brain insultus':ab,ti OR 'brain vascular accident':ab,ti OR 'cerebral apoplexia':ab,ti OR 'cerebral insult':ab,ti OR 'cerebral stroke':ab,ti OR 'cerebral vascular accident':ab,ti OR 'cerebral vascular insufficiency':ab,ti OR 'cerebro vascular accident':ab,ti OR 'cerebrovascular arrest':ab,ti OR 'cerebrovascular failure':ab,ti OR 'cerebrovascular injury':ab,ti OR 'cerebrovascular insufficiency':ab,ti OR 'cerebrovascular insult':ab,ti OR 'cerebrum vascular accident':ab,ti OR 'cryptogenic stroke':ab,ti OR 'cva':ab,ti OR 'insultus cerebralis':ab,ti OR 'ischaemic seizure':ab,ti OR 'ischemic seizure':ab,ti OR 'stroke':ab,ti OR 'thrombotic stroke':ab,ti OR 'cerebrovascular accident':ab,ti | 607214 |
| #3 | #1 OR #2 | 745602 |
| #4 | 'complex regional pain syndrome type i'/exp | 6448 |
| #5 | 'complex regional pain syndrome 1':ab,ti OR 'complex regional pain syndrome type 1':ab,ti OR 'crps 1':ab,ti OR 'crps i':ab,ti OR 'crps type 1':ab,ti OR 'crps type i':ab,ti OR 'crps-i':ab,ti OR 'neuralgic shoulder amyotrophy':ab,ti OR 'post-traumatic dystrophy':ab,ti OR 'posttraumatic dystrophy':ab,ti OR 'reflex sympathetic dystrophy':ab,ti OR 'shoulder arm syndrome':ab,ti OR 'shoulder hand syndrome':ab,ti OR 'shoulder-hand syndrome':ab,ti OR 'sympathetic dystrophy syndrome':ab,ti OR 'sympathetic reflex dystrophy':ab,ti OR 'complex regional pain syndrome type i':ab,ti | 4240 |
| #6 | #4 OR #5 | 7612 |
| #7 | 'fire dragon cupping':ab,ti OR 'fire dragon pot':ab,ti OR 'fire dragon jar':ab,ti OR 'fire dragon can':ab,ti OR 'fire dragon pot therapy':ab,ti OR 'huolong pot':ab,ti OR (huolong:ab,ti AND 'fire dragon':ab,ti AND cupping:ab,ti) OR 'huolong guan':ab,ti OR 'huo long cupping':ab,ti OR 'huo long guan therapy':ab,ti OR 'huo long cupping therapy':ab,ti OR 'huo-long cupping therapy':ab,ti OR 'huolong guan combined therapy':ab,ti OR 'huolong guan nursing':ab,ti OR 'comprehensive moxibustion with huolong cupping':ab,ti OR 'dragon jar comprehensive moxibustion':ab,ti OR 'tcm huolong guan therapy':ab,ti | 7 |
| #8 | #3 AND #6 AND #7 | 0 |

## 1.4 Search Strategy of Web of science

| # | Search Query | Results |
| --- | --- | --- |
| 1 | ‘cerebrovascular accident’ OR 'accident, cerebrovascular' OR 'acute cerebrovascular lesion' OR 'acute focal cerebral vasculopathy' OR 'acute stroke' OR 'apoplectic stroke' OR 'apoplexia' OR 'apoplexy' OR 'blood flow disturbance, brain' OR 'brain accident' OR 'brain attack' OR 'brain blood flow disturbance' OR 'brain insult' OR 'brain insultus' OR 'brain vascular accident' OR 'cerebral apoplexia' OR 'cerebral insult' OR 'cerebral stroke' OR 'cerebral vascular accident' OR 'cerebral vascular insufficiency' OR 'cerebro vascular accident' OR 'cerebrovascular arrest' OR 'cerebrovascular failure' OR 'cerebrovascular injury' OR 'cerebrovascular insufficiency' OR 'cerebrovascular insult' OR 'cerebrum vascular accident' OR 'cryptogenic stroke' OR 'CVA' OR 'insultus cerebralis' OR 'ischaemic seizure' OR 'ischemic seizure' OR 'stroke' OR 'thrombotic stroke' OR 'cerebrovascular accident' (Topic) | 791511 |
| 2 | ‘cerebrovascular accident’ OR 'accident, cerebrovascular' OR 'acute cerebrovascular lesion' OR 'acute focal cerebral vasculopathy' OR 'acute stroke' OR 'apolectic stroke' OR 'apoplexie' OR 'apoplexy' OR 'blood flow disturbance, brain' OR 'brain accident' OR 'brain attack' OR 'brain blood flow disturbance' OR 'brain insult' OR 'brain insultos' OR 'brain vascular accident' OR 'cerebral apoplexie' OR 'cerebral insult' OR 'cerebral stroke' OR 'cerebral vascular accident' OR 'cerebral vascular insufficiency' OR 'cerebro vascular accident' OR 'cerebrovascular arrest' OR 'cerebrovascular failure' OR 'cerebrovascular injury' OR 'cerebrovascular insufficiency' OR 'cerebrovascular insult' OR 'cerebrum vascular accident' OR 'cryptogenic stroke' OR 'CVA' OR 'insultos cerebrales' OR 'ischaemic seizure' OR 'ischemic seizure' OR 'stroke' OR 'thrombotic stroke' OR 'cerebrovascular accident' (Topic) | 791420 |
| 3 | ‘complex regional pain syndrome type I’ OR 'complex regional pain syndrome 1' OR 'complex regional pain syndrome type 1' OR 'CRPS 1' OR 'CRPS I' OR 'CRPS type 1' OR 'CRPS type I' OR 'CRPS-I' OR 'neuralgic shoulder amyotrophy' OR 'post-traumatic dystrophy' OR 'posttraumatic dystrophy' OR 'reflex sympathetic dystrophy' OR 'shoulder arm syndrome' OR 'shoulder hand syndrome' OR 'shoulder-hand syndrome' OR 'sympathetic dystrophy syndrome' OR 'sympathetic reflex dystrophy' OR 'complex regional pain syndrome type I' (Topic) | 12745 |
| 4 | 'fire dragon cupping' OR 'fire dragon pot' OR 'fire dragon jar' OR 'fire dragon can' OR 'fire dragon pot therapy' OR 'huolong pot' OR ‘huolong cupping’ OR 'fire dragon cupping ' OR 'huolong guan' OR 'huo long cupping' OR 'huo long guan therapy' OR 'huo long cupping therapy' OR 'huo-long cupping therapy' OR 'huolong guan combined therapy' OR 'huolong guan nursing' OR 'comprehensive moxibustion with huolong cupping' OR 'dragon jar comprehensive moxibustion' OR 'tcm huolong guan therapy' (Topic) | 69 |
| 5 | #2 AND #3 AND #4 | 0 |

## 1.5 Search Strategy of Scopus

| Search number | Query | Results |
| --- | --- | --- |
| 1 | TITLE-ABS-KEY ( "cerebrovascular accident" OR "accident, cerebrovascular" OR "acute cerebrovascular lesion" OR "acute focal cerebral vasculopathy" OR "acute stroke" OR "apoplectic stroke" OR "apoplexia" OR "apoplexy" OR "blood flow disturbance, brain" OR "brain accident" OR "brain attack" OR "brain blood flow disturbance" OR "brain insult" OR "brain insultus" OR "brain vascular accident" OR "cerebral apoplexia" OR "cerebral insult" OR "cerebral stroke" OR "cerebral vascular accident" OR "cerebral vascular insufficiency" OR "cerebro vascular accident" OR "cerebrovascular arrest" OR "cerebrovascular failure" OR "cerebrovascular injury" OR "cerebrovascular insufficiency" OR "cerebrovascular insult" OR "cerebrum vascular accident" OR "cryptogenic stroke" OR "CVA" OR "insultus cerebralis" OR "ischaemic seizure" OR "ischemic seizure" OR "stroke" OR "thrombotic stroke" OR "cerebrovascular accident" ) | 708546 |
| 2 | TITLE-ABS-KEY ( "complex regional pain syndrome type I" OR "complex regional pain syndrome 1" OR "complex regional pain syndrome type 1" OR "CRPS 1" OR "CRPS I" OR "CRPS type 1" OR "CRPS type I" OR "CRPS-I" OR "neuralgic shoulder amyotrophy" OR "post-traumatic dystrophy" OR "posttraumatic dystrophy" OR "reflex sympathetic dystrophy" OR "shoulder arm syndrome" OR "shoulder hand syndrome" OR "shoulder-hand syndrome" OR "sympathetic dystrophy syndrome" OR "sympathetic reflex dystrophy" OR "complex regional pain syndrome type I" ) | 6573 |
| 3 | TITLE-ABS-KEY ( "fire dragon cupping" OR "fire dragon pot" OR "fire dragon jar" OR "fire dragon can" OR "fire dragon pot therapy" OR "huolong pot" OR "huolong cupping" OR "fire dragon cupping " OR "huolong guan" OR "huo long cupping" OR "huo long guan therapy" OR "huo long cupping therapy" OR "huo-long cupping therapy" OR "huolong guan combined therapy" OR "huolong guan nursing" OR "comprehensive moxibustion with huolong cupping" OR "dragon jar comprehensive moxibustion" OR "tcm huolong guan therapy" ) | 7 |
| 4 | 1 AND 2 AND 3 | 0 |

## 1.6 Search Strategy of CINAHL

| S# | Query | Results |
| --- | --- | --- |
| S1 | SU ("Stroke") OR XB (“cerebrovascular accident” OR “accident, cerebrovascular” OR “acute cerebrovascular lesion” OR “acute focal cerebral vasculopathy” OR “acute stroke” OR “apoplectic stroke” OR “apoplexia” OR “apoplexy” OR “blood flow disturbance, brain” OR “brain accident” OR “brain attack” OR “brain blood flow disturbance” OR “brain insult” OR “brain insultus” OR “brain vascular accident” OR “cerebral apoplexia” OR “cerebral insult” OR “cerebral stroke” OR “cerebral vascular accident” OR “cerebral vascular insufficiency” OR “cerebro vascular accident” OR “cerebrovascular arrest” OR “cerebrovascular failure” OR “cerebrovascular injury” OR “cerebrovascular insufficiency” OR “cerebrovascular insult” OR “cerebrum vascular accident” OR “cryptogenic stroke” OR “CVA” OR “insultus cerebralis” OR “ischaemic seizure” OR “ischemic seizure” OR “stroke” OR “thrombotic stroke” OR “cerebrovascular accident”) | 154590 |
| S2 | XB (“complex regional pain syndrome type I” OR “complex regional pain syndrome 1” OR “complex regional pain syndrome type 1” OR “CRPS 1” OR “CRPS I” OR “CRPS type 1” OR “CRPS type I” OR “CRPS-I” OR “neuralgic shoulder amyotrophy” OR “post-traumatic dystrophy” OR “posttraumatic dystrophy” OR “reflex sympathetic dystrophy” OR “shoulder arm syndrome” OR “shoulder hand syndrome” OR “shoulder-hand syndrome” OR “sympathetic dystrophy syndrome” OR “sympathetic reflex dystrophy” OR “complex regional pain syndrome type I”) | 893 |
| S3 | XB (“fire dragon cupping” OR “fire dragon pot” OR “fire dragon jar” OR “fire dragon can” OR “fire dragon pot therapy” OR “huolong pot” OR “huolong cupping” OR “fire dragon cupping “ OR “huolong guan” OR “huo long cupping” OR “huo long guan therapy” OR “huo long cupping therapy” OR “huo-long cupping therapy” OR “huolong guan combined therapy” OR “huolong guan nursing” OR “comprehensive moxibustion with huolong cupping” OR “dragon jar comprehensive moxibustion” OR “tcm huolong guan therapy”) | 9 |
| S4 | S1 AND S2 AND S3 | 1 |

## 1.7 Search Strategy of Ovid

| Search number | Query | Results |
| --- | --- | --- |
| 1 | Stroke/ | 148672 |
| 2 | (stroke or strokes or cerebrovascular accident or cerebrovascular accidents or cerebralcerebral strokes or stroke, cerebral or strokes, cerebral or cerebrovascular apoplexy or apoplexy, cerebrovascular or vascular accident, brain or brain vascular accident or brain vascular accidents or vascular accidents, brain or cerebrovascularcerebrovascular strokes or stroke, cerebrovascular or strokes, cerebrovascular or apoplexy or cva or cvas or stroke, acute or acuteacute strokes or strokes, acute or cerebrovascular accident, acute or acute cerebrovascular accident or acute cerebrovascular accidents or cerebrovascular accidents, acute).ab,ti,kw. | 386002 |
| 3 | 1 or 2 | 401452 |
| 4 | Reflex Sympathetic Dystrophy/ | 3691 |
| 5 | (reflex sympathetic dystrophy or dystrophies, reflex sympathetic or dystrophy, reflex sympathetic or reflex sympathetic dystrophies or sympathetic dystrophies, reflex or sympathetic dystrophy, reflex or crps type is or type i, crps or complex regional pain syndrome, type i or pain syndrome type i, regional, complex or sudek atrophy or atrophy, sudek or reflex sympathetic dystrophy syndrome or rsd or rsds or sympathetic reflex dystrophia or reflex dystrophia, sympathetic or sympathetic reflex dystrophias or syndrome,type i complex regional pain syndrome or sudek's atrophy or atrophies, sudek's or atrophy, sudek's or sudek's atrophies or sudeks atrophy or pain syndrome type i, complex regional or algodystrophic syndrome or syndrome, algodystrophic or algodystrophy or algodystrophies or cervical sympathetic dystrophy or cervical sympathetic dystrophies or dystrophies, cervical sympathetic or dystrophy, cervical sympathetic or sympathetic dystrophies, cervical or sympathetic dystrophy, cervical or shoulder-hand syndrome or shoulder hand syndrome or shoulder-hand syndromes or syndrome, shoulder-hand or syndromes, shoulder-hand).ab,ti,kw. | 30827 |
| 6 | 4 or 5 | 32415 |
| 7 | (Fire Dragon Cupping or Fire Dragon Pot or Fire Dragon Jar or Fire Dragon Can or Fire Dragon Pot Therapy or Huolong Pot or Huolong Cupping or Huolong Guan or Huo Long Cupping or Huo Long Guan Therapy or Huo Long Cupping Therapy or Huo-Long Cupping Therapy or Huolong Guan Combined Therapy or Huolong Guan Nursing or Comprehensive Moxibustion With Huolong Cupping or Dragon Jar Comprehensive Moxibustion or Tcm Huolong Guan Therapy).ab,ti,kw. | 4 |
| 8 | 3 and 6 and 7 | 0 |

## 1.8 Search strategy of CNKI

| Search number | Query | Results |
| --- | --- | --- |
| 1 | (SU=卒中 + 急性卒中 + 脑卒中 + 脑中风 + 脑血管意外 + CVA + CVAs + 脑血管中风 + 中风，急性 + 急性脑血管意外 + 急性脑卒中 + 缺血性卒中) | 199481 |
| 2 | (SU=反射性交感神经营养障碍 + 肩手综合征 + 痛性营养不良综合征 + 痛性营养障碍 + 颈交感神经性营养不良 + RSD + RSDs + 反射性交感神经营养不良 + 复合性局部疼痛综合征Ⅰ型 + Sudek's萎缩 + Sudek萎缩 + 反射性交感神经营养障碍综合征) | 26458 |
| 3 | 1 AND 2 | 2166 |
| 4 | (SU=火龙罐 + 火龙罐疗法 + 火龙罐综合灸 + 火龙罐灸 + 火龙罐护理) | 499 |
| 5 | 3 AND 4 | 9 |

## 1.9 Search strategy of Wan Fang Data

| Search number | Query | Results |
| --- | --- | --- |
| 1 | 主题:(卒中 OR 急性卒中 OR 脑卒中 OR 脑中风 OR 脑血管意外 OR CVA OR CVAs OR 脑血管中风 OR 中风，急性 OR 急性脑血管意外 OR 急性脑卒中 OR 缺血性卒中) | 287927 |
| 2 | 主题:(反射性交感神经营养障碍 OR 肩手综合征 OR 痛性营养不良综合征 OR 痛性营养障碍 OR 颈交感神经性营养不良 OR RSD OR RSDs OR 反射性交感神经营养不良 OR 复合性局部疼痛综合征Ⅰ型 OR Sudek's萎缩 OR Sudek萎缩 OR 反射性交感神经营养障碍综合征) | 160938 |
| 3 | 1 AND 2 | 2465 |
| 4 | 主题: (火龙罐 OR 火龙罐疗法 OR 火龙罐综合灸 OR 火龙罐灸 OR 火龙罐护理) | 692 |
| 5 | 3 AND 4 | 15 |

## 1.10 Search strategy of VIP

| Search number | Query | Results |
| --- | --- | --- |
| 1 | U=(卒中 OR 急性卒中 OR 脑卒中 OR 脑中风 OR 脑血管意外 OR CVA OR CVAs OR 脑血管中风 OR 中风，急性 OR 急性脑血管意外 OR 急性脑卒中 OR 缺血性卒中) | 198827 |
| 2 | U=(反射性交感神经营养障碍 OR 肩手综合征 OR 痛性营养不良综合征 OR 痛性营养障碍 OR 颈交感神经性营养不良 OR反射性交感神经营养不良 OR 复合性局部疼痛综合征 OR反射性交感神经营养障碍综合征) | 3398 |
| 3 | 1 and 2 | 2025 |
| 4 | U=(火龙罐 OR 火龙罐疗法 OR 火龙罐综合灸 OR 火龙罐灸 OR 火龙罐护理) | 532 |
| 5 | 3 and 4 | 13 |

## 1.11 Search strategy of Sinomed

| Search number | Query | Results |
| --- | --- | --- |
| 1 | "卒中"[不加权:扩展] | 460384 |
| 2 | 急性卒中 OR 脑卒中 OR 脑中风 OR 脑血管意外 OR CVA OR CVAs OR 脑血管中风 OR 中风，急性 OR 急性脑血管意外 OR 急性脑卒中 OR 缺血性卒中 | 815568 |
| 3 | 1 OR 2 | 815568 |
| 4 | "反射性交感神经营养障碍"[不加权:扩展] | 6065 |
| 5 | 肩手综合征 OR 痛性营养不良综合征 OR 痛性营养障碍 OR 颈交感神经性营养不良 OR RSD OR RSDs OR 反射性交感神经营养不良 OR 复合性局部疼痛综合征Ⅰ型 OR Sudek's萎缩 OR Sudek萎缩 OR 反射性交感神经营养障碍综合征 | 110426 |
| 6 | 4 OR 5 | 110501 |
| 7 | 3 AND 6 | 2573 |
| 8 | 火龙罐 OR 火龙罐疗法 OR 火龙罐综合灸 OR 火龙罐灸 OR 火龙罐护理 | 418 |
| 9 | 7 AND 8 | 14 |

# 2 GRADE

| **Certainty assessment** | | | | | | | **№ of patients** | | **Effect** | | **Certainty** | **Importance** |
| --- | --- | --- | --- | --- | --- | --- | --- | --- | --- | --- | --- | --- |
| **№ of studies** | **Study design** | **Risk of bias** | **Inconsistency** | **Indirectness** | **Imprecision** | **Other considerations** | **FDC** | **o** | **Relative (95% CI)** | **Absolute (95% CI)** |  |  |
| **upper limb pain** | | | | | | | | | | | | |
| 10 | randomised trials | serious^a^ | very serious^b^ | serious^c^ | serious^d^ | none | 486 | 485 | - | SMD **1.35 SD lower** (2.13 lower to 2.93 lower) | ⨁◯◯◯ Very low^a,b,c,d^ |  |
| **motorfunction** | | | | | | | | | | | | |
| 10 | randomised trials | serious^a^ | not serious | serious^c^ | not serious | none | 486 | 485 | - | SMD **0.63 SD higher** (0.06 higher to 1.2 higher) | ⨁⨁◯◯ Low^a,c,d^ |  |
| **hand sweling** | | | | | | | | | | | | |
| 8 | randomised trials | serious^a^ | very serious^b^ | serious^c^ | not serious | none | 353 | 352 | - | SMD **1.01 SD lower** (1.94 lower to 0.09 lower) | ⨁◯◯◯ Very low^a,b,c^ |  |
| **Clinical efficacy** | | | | | | | | | | | | |
| 6 | randomised trials | serious^a^ | not serious | serious^c^ | not serious | none | 234/235 (99.6%) | 191/244 (78.3%) | **RR 1.20** (1.12 to 1.28) | **157 more per 1,000** (from 94 more to 219 more) | ⨁⨁◯◯ Low^a,c^ |  |

**CI:** confidence interval; **RR:** risk ratio; **SMD:** standardised mean difference

#### 2.1.1.1 Explanations

a. Lacking blinding

b. Substantial heterogeneity

c. All included trials and participants originated exclusively from a single country (China), which severely restricts the external validity and generalizability of the findings to non-Chinese populations.

d. The funnel chart is asymmetrical

**CI:** confidence interval; **RR:** risk ratio; **SMD:** standardised mean difference

# 3 Supplementary Figures

## 3.1 Subgroup Analysis

**Supplementary Figure 1.** Subgroup of Upper Motor Function

**Supplementary Figure 2.** Subgroup of Hand-Swelling

**Supplementary Figure 3.** Subgroup of Pain Intensity

| **outcome** | **Covariate** | **Estimate** | **95% CI** | ***P*-value** | **R^2^** |
| --- | --- | --- | --- | --- | --- |
| Pain intensity | Publication Year | -0.25 | [-0.84, 0.35] | 0.411 | 0.00% |
| Upper motor function | Publication Year | 0.12 | [-0.13, 0.36] | 0.355 | 0.00% |

The *R* package and its version used in this study

***R*** **version 4.5.1 (2025-06-13)**

**attached base packages:**

*grid, stats, graphics, grDevicesutils, datasets, methods, base.*

**other attached packages:**

*meta_8.2-1, metafor_4.8-0, numDeriv_2016.8-1.1, Matrix_1.7-4, stringr_1.6.0, dplyr_1.1.4, readxl_1.4.5, metadat_1.4-0.*

**loaded via a namespace (and not attached):**

*gtable_0.3.6, compiler_4.5.1, tidyselect_1.2.1, Rcpp_1.1.0, xml2_1.5.0, splines_4.5.1, scales_1.4.0, boot_1.3-32, lattice_0.22-7, readr_2.1.6, ggplot2_4.0.1, R6_2.6.1, generics_0.1.4, rbibutils_2.4, MASS_7.3-65, tibble_3.3.0, nloptr_2.2.1, minqa_1.2.8, tzdb_0.5.0, pillar_1.11.1, RColorBrewer_1.1-3, rlang_1.1.6, stringi_1.8.7, mathjaxr_1.8-0, S7_0.2.1, cli_3.6.5, magrittr_2.0.4, Rdpack_2.6.4, digest_0.6.39, rstudioapi_0.17.1, hms_1.1.4, lme4_1.1-37, lifecycle_1.0.4, CompQuadForm_1.4.4, nlme_3.1-168, reformulas_0.4.2, vctrs_0.6.5, glue_1.8.0, cellranger_1.1.0, farver_2.1.2, purrr_1.2.0, tools_4.5.1, pkgconfig_2.0.3.*
